# Craniovertebral Junction Deformity Diagnosed by Computed Tomography: A Case Report

**DOI:** 10.7759/cureus.67420

**Published:** 2024-08-21

**Authors:** Anjali Kumari, Gaurav V Mishra, Pratapsingh Parihar, Shivali V Kashikar, Sakshi S Dudhe, Rakshanda Agrawal, Paritosh N Bhangale

**Affiliations:** 1 Radiodiagnosis, Jawaharlal Nehru Medical College, Datta Meghe Institute of Higher Education and Research, Wardha, IND

**Keywords:** neurological symptoms, cervical spine ct, odontoid process herniation, atlanto-occipital assimilation, basilar invagination, craniovertebral junction deformity

## Abstract

Craniovertebral junction (CVJ) deformities, including basilar invagination and atlanto-occipital assimilation, present significant challenges in diagnosis and management due to their complex nature and impact on neurological function. We report a case of a 28-year-old female who experienced neck pain, weakness, tingling in the upper limbs, restricted neck movements, occipital headaches, and intermittent dizziness. These symptoms progressively worsened over six months, markedly affecting her quality of life. Neurological examination revealed reduced motor power in the upper limbs and a diminished bicipital tendon reflex, while other assessments remained normal. Cervical spine CT imaging was done which revealed basilar invagination and atlanto-occipital assimilation. This case underscores the importance of recognizing and managing CVJ deformities, highlighting the need for a multidisciplinary approach to address anatomical and associated neurological symptoms. Early and accurate diagnosis and a tailored treatment strategy are crucial for improving patient outcomes.

## Introduction

Craniovertebral junction (CVJ) deformities encompass a range of complex anomalies involving the base of the skull and the upper cervical spine [1]. Two notable conditions within this category are basilar invagination and atlanto-occipital assimilation. Understanding these deformities is crucial for effective diagnosis and treatment [2]. Basilar invagination refers to the herniation of the odontoid process of the axis (C2 vertebra) into the foramen magnum. This condition can cause significant neurological deficits due to compression of the brainstem and upper cervical spinal cord. It is often associated with various congenital conditions, including Chiari malformation and rheumatoid arthritis [3,4]. The severity of basilar invagination is typically assessed using radiographic measurements such as the position of the odontoid process relative to the Chamberlain line and the atlanto-dens interval (ADI) [5].

Atlanto-occipital assimilation involves the fusion of the atlas (C1 vertebra) with the occipital bone. This congenital anomaly can alter the biomechanics of the CVJ and may lead to clinical symptoms similar to those of basilar invagination, including neck pain and neurological deficits [6,7]. The condition can be associated with other craniovertebral anomalies and often requires careful radiological evaluation to determine the extent of fusion and its impact on the surrounding structures [8]. Together, these deformities can present a complex clinical picture, with symptoms ranging from neck pain and restricted movements to severe neurological impairment. Accurate diagnosis through imaging modalities such as CT and MRI are essential for appropriate management [9]. A multidisciplinary approach involving neurosurgery and orthopedics is often necessary to address anatomical deformities and neurological complications [10].

## Case presentation

A 28-year-old female presented to the clinic with a chief complaint of neck pain radiating to both shoulders, accompanied by weakness and tingling sensations in both upper limbs. These symptoms had been progressively worsening over the past six months. Additionally, the patient reported restricted neck movements, occipital headaches, and intermittent dizziness for approximately one year. Her symptoms had begun to interfere with her daily activities and overall quality of life. On neurological examination, the patient exhibited power of 4/5 in both upper limbs, with a noticeable reduction in the bicipital tendon reflex on the right side. Other neurological assessments, including lower limb strength and gait evaluation, were unremarkable despite significant symptoms. The physical findings suggested a possible CVJ anomaly.

**Figure 1:**
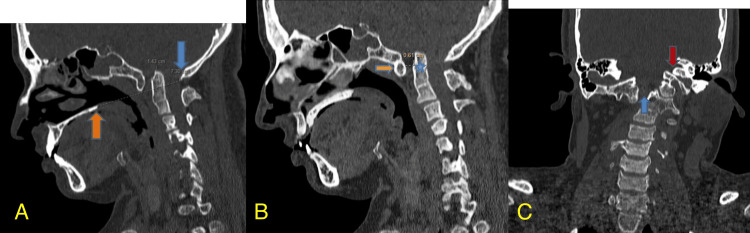
(A) Sagittal section of CT cervical spine showing basilar invagination. The tip of the odontoid process projects 14.3 mm above the Chamberlain line into the foramen magnum (orange and blue arrows indicate measurement points); (B) Sagittal section of CT cervical spine showing the atlanto-dens interval (ADI). The interval measures 6.1 mm, with the measurement points indicated by asterisks and arrows; (C) Coronal section of CT cervical spine showing non-visualization of the posterior arch of the atlas (C1 vertebra) with fusion of the posterior tubercle to the occiput (blue and red arrows indicate key structures).

To further investigate the underlying cause of her symptoms, a cervical spine CT scan was conducted. The imaging revealed several critical findings. There was evident basilar invagination, characterized by herniation of the odontoid process into the foramen magnum. The tip of the odontoid process was found to be 14.3 mm above the Chamberlain line, extending into the foramen magnum, which is significantly beyond the normal range. Additionally, the CT scan demonstrated atlanto-occipital assimilation, with non-visualization of the posterior arch of the atlas (C1 vertebra) and fusion of the posterior tubercle to the occiput. These findings were further corroborated by measuring the ADI, which was 6.1 mm, exceeding the normal range of 3.4 mm (Figure 1).

The combination of these imaging findings, along with the clinical presentation, pointed towards a complex CVJ deformity involving both basilar invagination and atlanto-occipital assimilation. The patient’s condition necessitates a comprehensive management plan, likely involving a multidisciplinary team to address both the anatomical deformities and the associated neurological symptoms.

## Discussion

CVJ deformities, such as basilar invagination and atlanto-occipital assimilation, present complex challenges in diagnosis and management. The patient in this case exhibited both conditions, leading to significant neurological and musculoskeletal symptoms [11]. Basilar Invagination occurs when the odontoid process herniates into the foramen magnum, often resulting in compression of the brainstem and upper spinal cord. This condition can manifest with symptoms such as neck pain, headaches, and neurological deficits, which are consistent with our patient’s presentation [12]. The herniation of the odontoid process was 14.3 mm above the Chamberlain line, indicative of severe basilar invagination. This degree of displacement can lead to significant brainstem compression, contributing to the patient’s symptoms [13].

Atlanto-occipital assimilation refers to the fusion of the atlas (C1 vertebra) with the occipital bone, which can alter the biomechanics of the CVJ and potentially exacerbate symptoms associated with other CVJ anomalies [14]. In our case, the non-visualization of the posterior arch of the atlas and the fusion to the occiput, coupled with an ADI of 6.1 mm, indicated a substantial alteration in the normal anatomy, which can impact spinal stability and lead to neurological impairment [15]. The combination of these deformities can complicate the clinical picture, leading to a range of symptoms from localized neck pain to more generalized neurological deficits [9]. Early diagnosis through imaging is crucial, as it allows for timely intervention to prevent further deterioration of neurological function [16]. Management of CVJ deformities often requires a multidisciplinary approach involving neurosurgeons, orthopedic surgeons, and radiologists. Surgical intervention may be necessary to decompress the spinal cord and stabilize the CVJ [17]. In this case, the patient's symptoms and imaging findings suggest the need for a detailed surgical plan tailored to address both the anatomical deformities and the associated neurological symptoms [18].

## Conclusions

In conclusion, this case underscores the complexity of managing CVJ deformities, particularly when both basilar invagination and atlanto-occipital assimilation are present. The combination of these anomalies presents a challenging clinical picture, as evidenced by the patient's progressive symptoms and significant radiological findings. The significant herniation of the odontoid process and fusion of the atlas to the occiput contribute to substantial neurological impairment and discomfort. Effective management requires a multidisciplinary approach, integrating neurosurgical, orthopedic, and radiological expertise to address anatomical abnormalities and the associated symptoms. Early and accurate diagnosis, followed by a tailored treatment plan, is crucial for optimizing patient outcomes and improving quality of life.
